# Dexmedetomidine as an Opioid-Sparing Agent in Pediatric Craniofacial Surgery

**DOI:** 10.3390/children7070068

**Published:** 2020-07-01

**Authors:** Srijaya K. Reddy, Jacob J. Jones, Heather Gordish-Dressman, Sophie R. Pestieau

**Affiliations:** 1 Department of Anesthesiology, Division of Pediatric Anesthesiology—Monroe Carell Jr. Children’s Hospital, Vanderbilt University Medical Center, 2200 Children’s Way Suite 3116, Nashville, TN 37232, USA; 2 Division of Anesthesiology, Pain and Perioperative Medicine—Children’s National Hospital, The George Washington University School of Medicine and Health Sciences, 111 Michigan Avenue NW, Washington, DC 20010, USA; jjones6@cnmc.org (J.J.J.); spestiea@cnmc.org (S.R.P.); 3 Center for Translational Research—Children’s National Hospital, The George Washington University School of Medicine and Health Sciences, 111 Michigan Avenue NW, Washington, DC 20010, USA; hgordish@cnmc.org

**Keywords:** craniosynostoses, pediatric, anesthesia, postoperative pain

## Abstract

Pediatric craniofacial reconstruction surgery is associated with significant perioperative analgesic requirements. As dexmedetomidine mediates central nervous system sympathetic activity and pain modulation, its intraoperative use could be beneficial in craniofacial surgery. We hypothesized that intraoperative administration of dexmedetomidine in children undergoing craniofacial reconstructive surgery would result in reduced opioid requirements, pain, sedation scores, and opioid-induced side effects compared to patients who did not receive dexmedetomidine. All patients who underwent craniofacial reconstructive surgery at our institution from July 2013 to June 2017 were retrospectively evaluated. The primary outcome measure was mean postoperative morphine equivalent requirements. Secondary outcome measures included incidence of opioid-related side effects, pain scores, and hospital length of stay. Thirty-nine patients received dexmedetomidine intraoperatively while 41 patients did not. There was no difference in postoperative opioid requirements or pain scores between the two cohorts. However, patients who received higher doses of dexmedetomidine (4.7 mcg/kg) intraoperatively exhibited significantly lower rescue medication requirements for nausea and vomiting postoperatively. Contrary to the hypothesis, dexmedetomidine was not associated with reduced postoperative opioid requirements or pain scores in children undergoing craniofacial reconstructive surgery. However, our findings do suggest that dexmedetomidine may be beneficial in reducing side effects such as postoperative nausea and vomiting. A randomized controlled trial would be necessary to verify these findings.

## 1. Introduction

Postoperative pain is a common challenge in the daily practice of anesthesia, and in many cases, continues to be inadequately managed. This is particularly true of pain in children due to difficulties with pain assessment and concerns related to side effects of opioids [1]. While opioids remain the mainstay for pain control after major surgery, their use can lead to multiple adverse effects, including nausea, vomiting, urinary retention, hypoventilation, ileus, prolonged hospital stay, and increased healthcare costs [2]. Perioperative pain control is particularly important in pediatrics since inadequate treatment has also been associated with the detrimental progression of pain perception and likelihood that chronic pain will develop in the future [3]. Perioperative clinicians thus tend to adopt a multimodal approach to perioperative pain control using non-opioid medications such as non-steroidal anti-inflammatory drugs, acetaminophen, local anesthetics, and alpha-2 receptor agonist as adjuncts [4].

Dexmedetomidine is a highly selective alpha-2 receptor agonist that mediates central nervous system sympathetic activity and pain modulation, particularly in the spinal cord dorsal root ganglia [5]. Dexmedetomidine has been widely utilized to provide sedation, anxiolysis, and analgesia without producing respiratory depression [6,7]. Its use as an opioid-sparing adjunct in various surgeries has led to mixed results [8,9,10,11,12].

Complex cranial vault reconstruction (CCVR) is extensive, often requiring wide scalp dissections and multiple osteotomies, and is associated with significant postoperative pain and analgesic requirements. The aim of this pilot study was to determine if dexmedetomidine is associated with reduced postoperative opioid requirements in infants and children undergoing CCVR. We hypothesized that children with craniosynostosis undergoing CCVR who received intraoperative dexmedetomidine as an adjunct to multimodal pain management would have decreased postoperative opioid requirements and opioid-related side effects compared to children who did not receive dexmedetomidine.

## 2. Materials and Methods

### 2.1. Study Population and Outcomes of Interest

This retrospective study was conducted after approval from the Children’s National Hospital Institutional Review Board on 24 October 2018, and need for patient or parental consent was waived. All patients with craniosynostosis who underwent CCVR between July 2013 and June 2017 were identified and included in the study. Demographic and perioperative data collected included age, weight, gender, race, ethnicity, American Society of Anesthesiologists (ASA) classification, craniofacial syndromic status, number of cranial sutures, operative time, pediatric intensive care unit (PICU) days, total hospital days, and mechanical ventilator days.

Postoperative outcomes of interest included pain scores and administration of opioids (converted to weight-based morphine equivalents), acetaminophen, ketorolac, benzodiazepines and dexmedetomidine in the first 24 h postoperatively. Need for rescue medications for opioid-related side effects as evidenced by administration of ondansetron, promethazine, diphenhydramine and/or naloxone was also noted. Mean pain scores and highest pain scores in the first 24 postoperative hours were recorded using the Faces Legs Activity Cry Consolability Revised (FLACC-R) Scale.

### 2.2. Statistical Analysis

Demographic characteristics of sex, race, ethnicity, ASA classification, number of sutures, and syndromic status were compared between those receiving dexmedetomidine and those who did not using a Fisher’s exact test. Age and weight, neither of which was normally distributed, were compared using a Wilcoxon rank sum test. Pain scores were compared using a Student’s *t*-test, morphine and acetaminophen were compared using a Wilcoxon rank sum test, whether or not ketorolac was administered was compared using a Fisher’s exact test, and lastly, the relationship between dexmedetomidine and need for rescue medications was determined using Poisson regression models with a goodness of fit test performed (if Poisson model was not an adequate fit, a negative binomial regression model was used). The statistically significant level was set a priori at 0.05 and all analyses were performed with STATA V15 (College Station, TX, USA).

## 3. Results

### 3.1. Patient Characteristics

Eighty patients with craniosynostosis underwent CCVR, with 39 patients receiving dexmedetomidine intraoperatively and 41 patients who did not receive any intraoperative dexmedetomidine. There was a significant difference in terms of sex between the two cohorts in that those receiving dexmedetomidine were more predominantly female than those that did not receive dexmedetomidine (Table 1). There were no other significant demographic differences observed between the two groups.

### 3.2. Intraoperative Practices

All 80 patients were taken care of by a craniofacial team anesthesiologist with minimal variation in intraoperative management or surgical technique. All patients underwent general endotracheal anesthesia, and all patients had local anesthetic infiltrated subcutaneously at the surgical incision site at the beginning of the operation. The use of dexmedetomidine intraoperatively, including dosage determination, was at the discretion of the anesthesiologist. No other analgesic medications were administered intraoperatively except for opioids. There was no difference in intraoperative administration of weight-based morphine equivalents (in mg/kg) between children who received intraoperative dexmedetomidine and those who did not (0.48 ± 0.2 vs. 0.49 ± 0.3, *p* = 0.9). There was no significant difference in operative times between the two groups or the number of cranial sutures involved in the surgical repair (Table 1).

### 3.3. Postoperative Outcomes

There was no difference in postoperative opioid requirements or pain scores when comparing the two cohorts (Table 2). Additionally, there was no significant difference in PICU days, total hospital days, or mechanical ventilator days between the two groups (Table 1). However, patients who received higher doses of intraoperative dexmedetomidine did have a significantly lower need for rescue medication administration for nausea and vomiting postoperatively. There was a significant relationship between the number of doses of ondansetron given and the amount of intraoperative dexmedetomidine administered (Table 3). This was true in all patients (*p* = 0.049) and when limited to only patients receiving intraoperative dexmedetomidine (*p* = 0.017). In each case, as the amount of dexmedetomidine administered increased (range = 0–4.7 mcg/kg for all patients), the number of ondansetron doses decreased. This model tells us that, in all patients, with each increase in dexmedetomidine of one unit, we can predict the number of ondansetron doses given postoperatively to decrease by 0.342. In only those receiving dexmedetomidine intraoperatively (range = 0.2–4.7 mcg/kg), with each increase in dexmedetomidine by one unit, we can predict the number of ondansetron doses administered to decrease by 0.789 (Table 3).

## 4. Discussion

This preliminary study suggests that intraoperative dexmedetomidine use during craniofacial reconstructive surgery is not associated with improved postoperative pain scores or reduced perioperative opioid requirements. This is contrary to our hypothesis and other studies, which have highlighted dexmedetomidine as an opioid-sparing agent. Despite the lack of difference in opioid requirement between the groups, the need for ondansetron appeared to be inversely related to dexmedetomidine use. There is also evidence to suggest that dexmedetomidine has antiemetic efficacy in other types of surgery [13,14,15].

This retrospective observational study has some important limitations, including small sample size and a demographic difference between the two groups studied. Those receiving dexmedetomidine were more likely to be female than those that did not receive dexmedetomidine. While it is a well-known concept that female gender is a reliable predictor of postoperative nausea and vomiting in adults [16], there is insufficient evidence to support that the same is true in infants and young children. Most of the pediatric data for postoperative nausea and vomiting are in children who are at least 3 years of age [17]. Similarly, there is evidence that adult women have increased pain sensitivity and risk for postoperative pain [18]; however, this same phenomenon has not been observed in infants and young children.

## 5. Conclusions

Dexmedetomidine was not associated with reduced postoperative opioid requirements or pain scores in children undergoing CCVR. These preliminary results highlight that opioid-sparing multimodal approaches to pain management, though preferable, are not always a panacea. Furthermore, as dexmedetomidine is a relatively expensive analgesic medication with potential added financial burden for patients, the lack of significant benefit may not warrant the additional cost. While dexmedetomidine may not be a beneficial adjunct for pain control in this setting, its relationship with a lower use of antiemetics needs to be further explored.

## Figures and Tables

**Table 1:** Demographics and other characteristics.

	Did Not Receive Dexmedetomidine			Received Dexmedetomidine			
	N (%)	Mean ± SD	Median (Min–Max)	N (%)	Mean ± SD	Median (Min–Max)	*p*-Value
Age (months)	41	32 ± 31	26 (5–144)	39	29 ± 31	16 (4–132)	0.78
Weight (kg)	41	15 ± 7	14.0 (6–36)	39	14 ± 8	10 (5–45)	0.06
Sex							0.03
Female	12 (29)			21 (54)			
Male	29 (71)			18 (46)			
Race							0.40
Asian	2 (5)			0 (0)			
Black	11 (27)			9 (23)			
Other	5 (12)			3 (8)			
White	23 (56)			27 (69)			
Ethnicity							0.10
Hispanic	8 (19)			14 (36)			
Non-Hispanic	33 (81)			25 (64)			
ASA classification							0.38
1	2 (5)			0 (0)			
2	27 (66)			17 (63)			
3	12 (29)			12 (31)			
Syndromic							0.81
No	35 (85)			34 (87)			
Yes	6 (15)			5 (13)			
Sutures							0.07
1	16 (39)			21 (54)			
2	3 (7)			7 (18)			
3	7 (17)			6 (15)			
≥4	15 (37)			5 (13)			
Operative time (minutes)	41	287 ± 103	265 (123, 555)	39	264 ± 84	252 (106, 588)	0.48
PICU length of stay (days)							0.35
2	37 (90)			31 (80)			
3	3 (7)			7 (18)			
4	1 (3)			1 (2)			
Total hospital length of stay (days)	41	4.2 ± 1.0	4 (2, 8)	39	4.0 ± 0.8	4 (3, 7)	0.30
Mechanical ventilation (days)							0.33
0	40 (98)			39 (100)			
1	1 (2)			0 (0)			

**Table 2:** Relationship between intraoperative dexmedetomidine administration and pain scores and opioid administration in the first 24 postoperative hours.

	Did Not Receive Dexmedetomidine			Received Dexmedetomidine			
	N (%)	Mean ± SD	Median (Min–Max)	N (%)	Mean ± SD	Median (Min–Max)	*p*-Value
Average postoperative pain score	41	2.7 ± 1.2	2.5 (0.7–5.3)	39	2.7 ± 1.3	2.6 (0.3–6.3)	0.99
Highest postoperative pain score	41	7.1 ± 0.3	7 (4–10)	39	7.2 ± 2.1	7 (2–10)	0.90
Postoperative morphine equivalent administration (mg/kg)	41	0.4 ± 0.3	0.31 (0–1.2)	39	0.3 ± 0.2	0.3 (0–0.8)	0.70
Postoperative acetaminophen administration (mg/kg)	41	58 ± 19	61 (0–87)	39	56 ± 17	60 (15–90)	0.24
Postoperative ketorolac administered							0.78
No	36 (88)			35 (90)			
Yes	5 (12)			4 (10)			

**Table 3:** Prediction model for relationship between the need for postoperative rescue medication and intraoperative dexmedetomidine use.

Cohort	Rescue Medication	N	Coefficient	95% CI	*p*-Value
**Including all patients (those who did not receive dexmedetomidine have a value of zero)**	ondansetron	80	−0.342 ^†^	−0.682–0.002	0.049
	promethazine	80	−1.467	−5.345–2.411	0.46
	benzodiazepine	80	0.198	−0.514–0.910	0.59
	diphenhydramine	80	−79.408	−44605–44445	0.99
	naloxone	80	−79.302	−89130–88971	0.99
**Including only patients who received dexmedetomidine**	ondansetron	39	−0.789 ^†^	−1.438–0.140	0.017
	promethazine	39	−8.067	−22.139–6.005	0.26
	benzodiazepine	39	−0.060	−1.177–1.057	0.92
	diphenhydramine	39			N/A *
	naloxone	39			N/A *

^†^ With each increase in dexmedetomidine by one unit, the need for ondansetron decreases by the absolute value of the coefficient; * All patients who received dexmedetomidine had zero doses of diphenhydramine or naloxone.
